# Poly[bis­(μ_2_-pyrazine-2-carboxyl­ato)-κ^3^ *N* ^1^,*O*:*O*′;κ^3^ *N* ^1^,*O*:*O*-cadmium(II)]

**DOI:** 10.1107/S1600536809053525

**Published:** 2009-12-24

**Authors:** Ge Liu

**Affiliations:** a Chifeng University, Chifeng 024000, People’s Republic of China

## Abstract

In the structure of the title compound, [Cd(C_5_H_3_N_2_O_2_)_2_]_*n*_, the Cd^II^ ion is six-coordinated by two N atoms and four O atoms from three different pyrazine-2-carboxyl­ate ligands. One N atom and one O atom of the carboxyl­ate group in the ligand coordinate to the metal center, forming a five-membered chelate ring. The carboxyl­ate anion adopts two types of bridging modes, *viz*. μ_2_-O and *syn*–*anti*. Two Cd^II^ ions form a centrosymmetric dimer *via* a μ_2_-O bridge, and the dimers are linked through the *syn*–*anti* carboxyl­ate functional group, forming a two-dimensional polymeric structure extending along (100).

## Related literature

The title compound is isostructural with the Mn(II) complex, see: Cai *et al.* (2002 ▶); Devereux *et al.* (2002 ▶); Liang *et al.* (2002 ▶). For a homologous Cd(II) complex with a picolinate ligand, see: Deloume & Loiseleur (1974 ▶). 
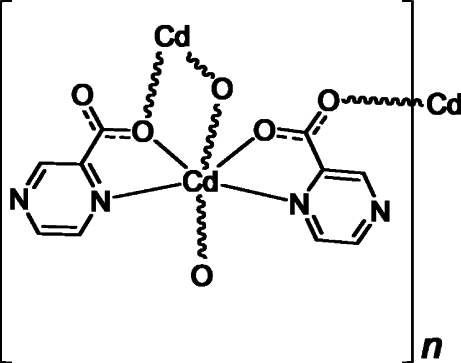


## Experimental

### 

#### Crystal data

[Cd(C_5_H_3_N_2_O_2_)_2_]
*M* *_r_* = 358.59
Monoclinic, 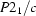

*a* = 10.304 (2) Å
*b* = 11.044 (2) Å
*c* = 10.274 (2) Å
β = 107.89 (3)°
*V* = 1112.7 (4) Å^3^
*Z* = 4
Mo *K*α radiation
μ = 1.98 mm^−1^
*T* = 293 K
0.18 × 0.13 × 0.12 mm

#### Data collection

Rigaku R-AXIS RAPID-S diffractometer
11295 measured reflections
2550 independent reflections
1943 reflections with *I* > 2σ(*I*)
*R*_int_ = 0.069

#### Refinement

*R*[*F* ^2^ > 2σ(*F* ^2^)] = 0.048
*wR*(*F* ^2^) = 0.065
*S* = 1.16
2550 reflections
172 parameters
H-atom parameters constrained
Δρ_max_ = 0.80 e Å^−3^
Δρ_min_ = −0.64 e Å^−3^

### 

Data collection: *RAPID-AUTO* (Rigaku, 1998 ▶); cell refinement: *RAPID-AUTO*; data reduction: *CrystalStructure* (Rigaku/MSC, 2002 ▶); program(s) used to solve structure: *SHELXS97* (Sheldrick, 2008 ▶); program(s) used to refine structure: *SHELXL97* (Sheldrick, 2008 ▶); molecular graphics: *SHELXTL* (Sheldrick, 2008 ▶); software used to prepare material for publication: *SHELXL97*.

## Supplementary Material

Crystal structure: contains datablocks I, global. DOI: 10.1107/S1600536809053525/bh2262sup1.cif

Structure factors: contains datablocks I. DOI: 10.1107/S1600536809053525/bh2262Isup2.hkl

Additional supplementary materials:  crystallographic information; 3D view; checkCIF report

## Figures and Tables

**Fig. 1.**
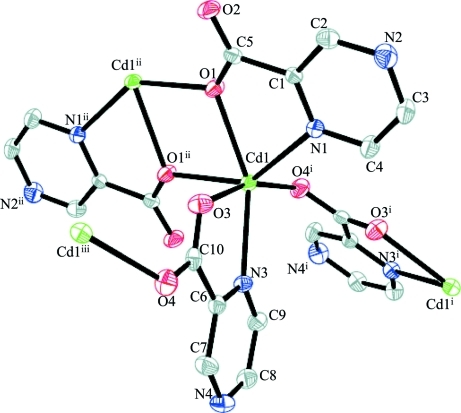
A view of the title compound showing the coordination of Cd atom with the atom-labelling scheme. Ellipsoids are drawn at the 30% probability level. H atoms have been omitted for clarity. [Symmetry codes: (i) x, -y + 3/2, z + 1/2; (ii) -x, -y + 1, -z; (iii) x, -y + 3/2, z

## supplementary crystallographic information

### Comment

Pyrazine-2-carboxylate is a suitable multidentate bridging ligand to build coordination polymers and several novel structural coordination polymers containing derivatives of pyrazinecarboxylate have been obtained. Here, we report the reaction of pyrazine-2-carboxylate with a Cd^II^ salt, which afforded a two-dimensional Cd^II^ coordination polymer. The compound is isostructural with the Mn^II^ complex (Cai *et al.*, 2002; Devereux *et al.*, 2002; Liang *et al.*, 2002). An isostructural Cd(II) complex including picolinate in place of pyrazine-2-carboxylate has also been X-ray characterized (Deloume & Loiseleur, 1974).

In the structure of the title compound, each Cd^II^ ion is six-coordinated by two N atoms and four O atoms from three different pyrazine-2-carboxylate ligands (Fig. 1). One N atom and one O atom of the neighboring carboxylate of the ligand coordinate to the center Cd^II^ ion forming a five-member chelate ring. The carboxylate of the ligand adopts two types of bridging mode: µ_2_-O and *syn*–*anti*. The second N atom is not involved in bonding, so the complex is also isostructural with the Cd(II) complex with picolinate (Deloume & Loiseleur, 1974). Cd^II^ ion form a dimer *via* an µ_2_-O bridge, and these dimers are linked through *syn*–*anti* carboxy O atoms, to form a two-dimensional structure.

### Experimental

A mixture of cadmium(II) nitrate (1 mmol), pyrazine-2-carboxylate (0.5 mmol), NaOH (1 mmol), tetrazolate (0.5 mmol) and water (10 ml) was sealed in a 23 ml Teflon-lined reactor, heated to 433 K at 10.8 K/h and kept at this temperature for three days, finally cooled to 303 K at 5.4 K/h (yield 20%).

### Refinement

Hydrogen atoms were included in calculated positions and treated as riding on their parent C atoms with C—H = 0.93 Å and *U*_iso_(H) = 1.2*U*_eq_(C).

### Crystal data

**Table:**

[Cd(C_5_H_3_N_2_O_2_)_2_]	*F*(000) = 696
*M**_r_* = 358.59	*D*_x_ = 2.141 Mg m^−^^3^
Monoclinic, *P*2_1_/*c*	Mo *K*α radiation, λ = 0.71073 Å
Hall symbol: -P 2ybc	Cell parameters from 9922 reflections
*a* = 10.304 (2) Å	θ = 3.1–27.5°
*b* = 11.044 (2) Å	µ = 1.98 mm^−^^1^
*c* = 10.274 (2) Å	*T* = 293 K
β = 107.89 (3)°	Block, colourless
*V* = 1112.7 (4) Å^3^	0.18 × 0.13 × 0.12 mm
*Z* = 4	

### Data collection

**Table:**

Rigaku R-AXIS RAPID-S diffractometer	1943 reflections with *I* > 2σ(*I*)
Radiation source: fine-focus sealed tube	*R*_int_ = 0.069
graphite	θ_max_ = 27.5°, θ_min_ = 3.1°
ω scans	*h* = −13→13
11295 measured reflections	*k* = −14→14
2550 independent reflections	*l* = −13→13

### Refinement

**Table:**

Refinement on *F*^2^	Primary atom site location: structure-invariant direct methods
Least-squares matrix: full	Secondary atom site location: difference Fourier map
*R*[*F*^2^ > 2σ(*F*^2^)] = 0.048	Hydrogen site location: inferred from neighbouring sites
*wR*(*F*^2^) = 0.065	H-atom parameters constrained
*S* = 1.16	*w* = 1/[σ^2^(*F*_o_^2^) + (0.0199*P*)^2^ + 0.0187*P*] where *P* = (*F*_o_^2^ + 2*F*_c_^2^)/3
2550 reflections	(Δ/σ)_max_ = 0.001
172 parameters	Δρ_max_ = 0.80 e Å^−^^3^
0 restraints	Δρ_min_ = −0.64 e Å^−^^3^

### Fractional atomic coordinates and isotropic or equivalent isotropic displacement parameters (Å^2^)

**Table:**

	*x*	*y*	*z*	*U*_iso_*/*U*_eq_	
C1	0.3232 (4)	0.6168 (3)	0.1469 (4)	0.0246 (9)	
C2	0.4605 (4)	0.6443 (4)	0.1809 (4)	0.0373 (10)	
H2	0.5148	0.6004	0.1402	0.045*	
C3	0.4363 (5)	0.7903 (4)	0.3276 (4)	0.0398 (12)	
H3	0.4728	0.8507	0.3915	0.048*	
C4	0.2983 (5)	0.7643 (4)	0.2953 (4)	0.0377 (11)	
H4	0.2446	0.8077	0.3370	0.045*	
C5	0.2594 (4)	0.5175 (4)	0.0442 (4)	0.0283 (10)	
C6	−0.1395 (4)	0.8772 (4)	−0.0343 (4)	0.0294 (10)	
C7	−0.2299 (5)	0.9723 (4)	−0.0745 (5)	0.0415 (12)	
H7	−0.2229	1.0226	−0.1446	0.050*	
C8	−0.3322 (5)	0.9200 (4)	0.0826 (5)	0.0412 (12)	
H8	−0.3991	0.9315	0.1250	0.049*	
C9	−0.2419 (4)	0.8262 (4)	0.1254 (4)	0.0376 (12)	
H9	−0.2484	0.7770	0.1967	0.045*	
C10	−0.0370 (4)	0.8481 (4)	−0.1094 (4)	0.0303 (10)	
Cd1	0.00719 (3)	0.63837 (3)	0.10693 (3)	0.02760 (11)	
N1	0.2415 (3)	0.6773 (3)	0.2048 (3)	0.0294 (8)	
N2	0.5188 (4)	0.7323 (4)	0.2710 (4)	0.0438 (10)	
N3	−0.1448 (3)	0.8035 (3)	0.0671 (3)	0.0298 (8)	
N4	−0.3279 (4)	0.9948 (4)	−0.0163 (4)	0.0473 (11)	
O1	0.1363 (3)	0.4919 (2)	0.0364 (3)	0.0345 (7)	
O2	0.3285 (3)	0.4700 (3)	−0.0196 (3)	0.0416 (8)	
O3	0.0344 (3)	0.7562 (3)	−0.0752 (3)	0.0396 (8)	
O4	−0.0375 (3)	0.9214 (3)	−0.2042 (3)	0.0411 (8)	

### Atomic displacement parameters (Å^2^)

**Table:**

	*U*^11^	*U*^22^	*U*^33^	*U*^12^	*U*^13^	*U*^23^
C1	0.023 (2)	0.027 (2)	0.024 (2)	0.0016 (18)	0.0073 (17)	0.0029 (18)
C2	0.032 (2)	0.039 (3)	0.044 (3)	0.001 (2)	0.016 (2)	0.001 (2)
C3	0.042 (3)	0.044 (3)	0.033 (3)	−0.009 (2)	0.010 (2)	−0.011 (2)
C4	0.036 (3)	0.042 (3)	0.038 (3)	−0.008 (2)	0.015 (2)	−0.011 (2)
C5	0.026 (2)	0.027 (2)	0.030 (3)	0.005 (2)	0.006 (2)	0.003 (2)
C6	0.028 (2)	0.032 (3)	0.028 (2)	−0.003 (2)	0.0085 (18)	−0.003 (2)
C7	0.043 (3)	0.040 (3)	0.042 (3)	0.008 (2)	0.013 (2)	0.011 (2)
C8	0.034 (3)	0.053 (3)	0.040 (3)	0.005 (2)	0.017 (2)	−0.007 (3)
C9	0.039 (3)	0.042 (3)	0.035 (3)	0.004 (2)	0.017 (2)	−0.003 (2)
C10	0.026 (2)	0.039 (3)	0.025 (2)	−0.013 (2)	0.0066 (19)	−0.006 (2)
Cd1	0.02236 (16)	0.02999 (17)	0.03203 (18)	0.00188 (16)	0.01067 (12)	−0.00474 (17)
N1	0.029 (2)	0.029 (2)	0.029 (2)	0.0000 (16)	0.0077 (17)	−0.0018 (16)
N2	0.035 (2)	0.052 (2)	0.045 (2)	−0.008 (2)	0.013 (2)	−0.004 (2)
N3	0.026 (2)	0.034 (2)	0.032 (2)	0.0025 (16)	0.0122 (17)	−0.0026 (17)
N4	0.039 (2)	0.048 (3)	0.057 (3)	0.015 (2)	0.016 (2)	0.000 (2)
O1	0.0240 (16)	0.0357 (17)	0.045 (2)	−0.0046 (14)	0.0130 (14)	−0.0159 (15)
O2	0.0314 (18)	0.049 (2)	0.049 (2)	0.0041 (15)	0.0189 (16)	−0.0136 (16)
O3	0.0391 (19)	0.040 (2)	0.0485 (19)	0.0050 (15)	0.0268 (16)	0.0049 (16)
O4	0.048 (2)	0.0451 (19)	0.0348 (18)	−0.0053 (16)	0.0193 (16)	0.0032 (16)

### Geometric parameters (Å, °)

**Table:**

C1—N1	1.348 (5)	C7—H7	0.9300
C1—C2	1.383 (5)	C8—N4	1.321 (6)
C1—C5	1.523 (5)	C8—C9	1.372 (6)
C2—N2	1.350 (5)	C8—H8	0.9300
C2—H2	0.9300	C9—N3	1.339 (5)
C3—N2	1.332 (5)	C9—H9	0.9300
C3—C4	1.388 (6)	C10—O3	1.240 (5)
C3—H3	0.9300	C10—O4	1.265 (5)
C4—N1	1.341 (5)	Cd1—O4^i^	2.227 (3)
C4—H4	0.9300	Cd1—O1^ii^	2.256 (3)
C5—O2	1.224 (4)	Cd1—O1	2.346 (3)
C5—O1	1.277 (4)	Cd1—N1	2.352 (3)
C6—N3	1.336 (5)	Cd1—N3	2.356 (3)
C6—C7	1.380 (5)	Cd1—O3	2.366 (3)
C6—C10	1.522 (5)	O1—Cd1^ii^	2.256 (3)
C7—N4	1.346 (5)	O4—Cd1^iii^	2.227 (3)
			
N1—C1—C2	120.6 (4)	O4—C10—C6	114.6 (4)
N1—C1—C5	117.8 (3)	O4^i^—Cd1—O1^ii^	96.34 (11)
C2—C1—C5	121.6 (4)	O4^i^—Cd1—O1	110.68 (11)
N2—C2—C1	122.5 (4)	O1^ii^—Cd1—O1	71.28 (11)
N2—C2—H2	118.8	O4^i^—Cd1—N1	98.14 (11)
C1—C2—H2	118.8	O1^ii^—Cd1—N1	140.96 (11)
N2—C3—C4	122.7 (4)	O1—Cd1—N1	69.70 (10)
N2—C3—H3	118.6	O4^i^—Cd1—N3	94.39 (11)
C4—C3—H3	118.6	O1^ii^—Cd1—N3	96.46 (11)
N1—C4—C3	120.7 (4)	O1—Cd1—N3	152.84 (11)
N1—C4—H4	119.6	N1—Cd1—N3	118.22 (11)
C3—C4—H4	119.6	O4^i^—Cd1—O3	163.44 (10)
O2—C5—O1	126.9 (4)	O1^ii^—Cd1—O3	92.72 (11)
O2—C5—C1	118.7 (4)	O1—Cd1—O3	85.35 (10)
O1—C5—C1	114.3 (4)	N1—Cd1—O3	83.22 (11)
N3—C6—C7	120.8 (4)	N3—Cd1—O3	70.77 (11)
N3—C6—C10	117.8 (4)	C4—N1—C1	117.6 (4)
C7—C6—C10	121.2 (4)	C4—N1—Cd1	126.8 (3)
N4—C7—C6	122.5 (4)	C1—N1—Cd1	114.8 (2)
N4—C7—H7	118.8	C3—N2—C2	115.9 (4)
C6—C7—H7	118.8	C6—N3—C9	116.7 (4)
N4—C8—C9	122.7 (4)	C6—N3—Cd1	115.1 (3)
N4—C8—H8	118.7	C9—N3—Cd1	128.0 (3)
C9—C8—H8	118.7	C8—N4—C7	115.7 (4)
N3—C9—C8	121.6 (4)	C5—O1—Cd1^ii^	128.4 (3)
N3—C9—H9	119.2	C5—O1—Cd1	118.3 (3)
C8—C9—H9	119.2	Cd1^ii^—O1—Cd1	108.72 (11)
O3—C10—O4	127.1 (4)	C10—O3—Cd1	118.1 (3)
O3—C10—C6	118.2 (4)	C10—O4—Cd1^iii^	121.9 (3)
			
N1—C1—C2—N2	−0.7 (6)	C8—C9—N3—Cd1	−173.5 (3)
C5—C1—C2—N2	179.6 (4)	O4^i^—Cd1—N3—C6	172.6 (3)
N2—C3—C4—N1	0.6 (7)	O1^ii^—Cd1—N3—C6	−90.5 (3)
N1—C1—C5—O2	172.7 (4)	O1—Cd1—N3—C6	−29.6 (4)
C2—C1—C5—O2	−7.6 (6)	N1—Cd1—N3—C6	70.9 (3)
N1—C1—C5—O1	−8.3 (5)	O3—Cd1—N3—C6	0.2 (3)
C2—C1—C5—O1	171.4 (4)	O4^i^—Cd1—N3—C9	−13.4 (3)
N3—C6—C7—N4	−0.9 (7)	O1^ii^—Cd1—N3—C9	83.5 (3)
C10—C6—C7—N4	175.2 (4)	O1—Cd1—N3—C9	144.4 (3)
N4—C8—C9—N3	−1.2 (7)	N1—Cd1—N3—C9	−115.0 (3)
N3—C6—C10—O3	1.6 (6)	O3—Cd1—N3—C9	174.2 (4)
C7—C6—C10—O3	−174.6 (4)	C9—C8—N4—C7	0.9 (7)
N3—C6—C10—O4	179.9 (3)	C6—C7—N4—C8	0.1 (7)
C7—C6—C10—O4	3.7 (6)	O2—C5—O1—Cd1^ii^	−5.3 (6)
C3—C4—N1—C1	0.0 (6)	C1—C5—O1—Cd1^ii^	175.9 (2)
C3—C4—N1—Cd1	−169.2 (3)	O2—C5—O1—Cd1	−158.4 (3)
C2—C1—N1—C4	0.1 (6)	C1—C5—O1—Cd1	22.8 (4)
C5—C1—N1—C4	179.8 (3)	O4^i^—Cd1—O1—C5	−112.2 (3)
C2—C1—N1—Cd1	170.6 (3)	O1^ii^—Cd1—O1—C5	158.0 (4)
C5—C1—N1—Cd1	−9.7 (4)	N1—Cd1—O1—C5	−20.9 (3)
O4^i^—Cd1—N1—C4	−66.6 (3)	N3—Cd1—O1—C5	91.6 (3)
O1^ii^—Cd1—N1—C4	−177.3 (3)	O3—Cd1—O1—C5	63.6 (3)
O1—Cd1—N1—C4	−175.7 (4)	O4^i^—Cd1—O1—Cd1^ii^	89.81 (14)
N3—Cd1—N1—C4	32.9 (4)	O1^ii^—Cd1—O1—Cd1^ii^	0.0
O3—Cd1—N1—C4	96.7 (3)	N1—Cd1—O1—Cd1^ii^	−178.94 (15)
O4^i^—Cd1—N1—C1	124.0 (3)	N3—Cd1—O1—Cd1^ii^	−66.4 (3)
O1^ii^—Cd1—N1—C1	13.3 (4)	O3—Cd1—O1—Cd1^ii^	−94.46 (13)
O1—Cd1—N1—C1	14.9 (3)	O4—C10—O3—Cd1	−179.5 (3)
N3—Cd1—N1—C1	−136.6 (3)	C6—C10—O3—Cd1	−1.5 (5)
O3—Cd1—N1—C1	−72.7 (3)	O4^i^—Cd1—O3—C10	−26.6 (5)
C4—C3—N2—C2	−1.1 (7)	O1^ii^—Cd1—O3—C10	96.6 (3)
C1—C2—N2—C3	1.2 (6)	O1—Cd1—O3—C10	167.6 (3)
C7—C6—N3—C9	0.6 (6)	N1—Cd1—O3—C10	−122.3 (3)
C10—C6—N3—C9	−175.6 (3)	N3—Cd1—O3—C10	0.7 (3)
C7—C6—N3—Cd1	175.3 (3)	O3—C10—O4—Cd1^iii^	31.3 (5)
C10—C6—N3—Cd1	−0.9 (4)	C6—C10—O4—Cd1^iii^	−146.8 (3)
C8—C9—N3—C6	0.4 (6)		

Symmetry codes: (i) *x*, −*y*+3/2, *z*+1/2; (ii) −*x*, −*y*+1, −*z*; (iii) *x*, −*y*+3/2, *z*−1/2.
